# 

**Published:** 1899-09-23

**Authors:** 


### NOTICE TO CORRESPONDENTS.

All MS., Letters, Books for Review, and other matters intended for the Editor should be addressed The Editor, "The Hospital," The Hospital Building, 28 & 29, Southampton Street, Strand, W.C.

The Editor cannot undertake to return rejected MS., even when accompanied by stamped directed envelope.

**Notice to Advertisers and Subscribers.**

All Advertisements, Orders for Copies of The Hospital, and Business Communications should be addressed to **THE MANAGER (Not to the Editor),** The Hospital, The Hospital Building, 28 & 29, Southampton Street, Strand, London, W.C.

The Cost of Subscription, post free, is as follows:—Per week, 2½d.; per quarter, 2s. 8d.; per half-year, 5s. 3d.; per annum, 10s. 6d. Foreign:—Per annum, 15s. 2d., payable, in all cases, in advance.

**NOTICE.—Vol. XXV. of "The Hospital" (October, 1898, to March, 1899), in handsome cloth binding, is now on sale at the Office of this Journal, price 7s. 6d. Cases for binding the Numbers contained in this Volume 1s. 6d. Index to Vol. XXV., 2½d., post free.**

**The Monthly Part for October will be ready on October 2nd. Price 6d., by post 8d.**

### Scraps and Gleanings.

The income last year of the North Staffordshire Infirmary amounted to £12,194 7s. 1d., and the expenditure to £12,376 1s. 11d.

The total amount collected this year in aid of the Scarborough Hospital and Dispensary on Hospital Saturday was £280 odd.

The total amount of contributions from workpeople during the past year to the Walsall and District Hospital is £1,283 5s., as compared with £1,172 the previous year.

A legacy of £100 to the Endowment Fund of the West Bromwich Hospital has been left by the late Mr. Hall, which amount now raises the amount invested to £5,300.

The Friendly Societies in Eastbourne and District are endeavouring to raise a fund for the erection of a Friendly Societies' Ward at the Princess Alice Memorial Hospital.

There have been 470 patients passing through Heatherdene Convalescent Home during the last year, the expenditure during which period being £748, as against £690 the previous year.

The work of installing the electric light at the Dundee Royal Infirmary is now almost completed. The improvements to the kitchen, which have been completed some little time, are found in practice to be most useful and helpful.

The amount received from the Hospital Saturday collections in Sydney, New South Wales, during 1898-99 was £3,885 18s. 4d., whilst the total collected since 1893-4, when the fund was first established, now amounts to £20,398.

Two hundred and seventy-five more out-patients passed through the Bristol General Hospital during the past year than were treated the previous year. The out-patients' department is to be reconstructed as soon as possible. Meanwhile, a balance of £350 remains to be contributed towards the cost of the new museum—£1,350 in all—of which sum £1,000 was given anonymously; and it is proposed to spend £500 on a reconstruction of the operation theatre.

### The Student of Medicine.

### APPOINTMENTS.

J. Brown, M.D., D.P.H.Vict.Univ., appointed Medical Officer of Health for the Borough of Bacup; also Physician to Sourhall Hospital. A. G. B. Lory, L.S.A Lond., appointed Medical Officer and Public Vaccinator for the Chudleigh District of the Newton Abbot Union. F. E. Taylor, M.A., M.B., M Sc.Vict., M.R.C.S.Eng, L.R.C.P.Lond., appointed Assistant Resident Medical Officer at Queen Charlotte's Lying in Hospital. T. W. Tetley, M.R.C.S., L.R.C.P., appointed House Physician to the Leeds General Infirmary. A. G. Wilson, B.A., M.B.Cambs., M.R.C.S., L.R.C.P.Lond., appointed House Surgeon to St. Mary's Hospital, London, W.

